# New regulatory thinking is needed for AI-based personalised drug and cell therapies in precision oncology

**DOI:** 10.1038/s41698-024-00517-w

**Published:** 2024-01-30

**Authors:** Bouchra Derraz, Gabriele Breda, Christoph Kaempf, Franziska Baenke, Fabienne Cotte, Kristin Reiche, Ulrike Köhl, Jakob Nikolas Kather, Deborah Eskenazy, Stephen Gilbert

**Affiliations:** 1 ProductLife Group, Paris, France; 2 Groupe de recherche et d’accueil en droit et économie de la santé (GRADES), Faculty of Pharmacy, University Paris-Saclay, Paris, France; 3 Fraunhofer Institute for Cell Therapy and Immunology, Leipzig, Germany; 4 Carl Gustav Carus University Hospital Dresden, Dresden University of Technology, Dresden, Germany; 5 Department of Emergency Medicine, University Clinic Marburg, Philipps-University, Marburg, Germany; 6 Center for Scalable Data Analytics and Artificial Intelligence (ScaDS.AI), Dresden/Leipzig, Germany; 7 Institute of Clinical Immunology, University Leipzig, Leipzig, Germany; 8 Else Kröner Fresenius Center for Digital Health, TUD Dresden University of Technology, Dresden, Germany

**Keywords:** Health policy, Drug regulation, Stem-cell therapies

## Abstract

Until recently the application of artificial intelligence (AI) in precision oncology was confined to activities in drug development and had limited impact on the personalisation of therapy. Now, a number of approaches have been proposed for the personalisation of drug and cell therapies with AI applied to therapy design, planning and delivery at the patient’s bedside. Some drug and cell-based therapies are already tuneable to the individual to optimise efficacy, to reduce toxicity, to adapt the dosing regime, to design combination therapy approaches and, preclinically, even to personalise the receptor design of cell therapies. Developments in AI-based healthcare are accelerating through the adoption of foundation models, and generalist medical AI models have been proposed. The application of these approaches in therapy design is already being explored and realistic short-term advances include the application to the personalised design and delivery of drugs and cell therapies. With this pace of development, the limiting step to adoption will likely be the capacity and appropriateness of regulatory frameworks. This article explores emerging concepts and new ideas for the regulation of AI-enabled personalised cancer therapies in the context of existing and in development governance frameworks.

## Introduction

There is a regulatory approval bottleneck in the translation of the latest advances in precision cancer therapies to patients^1^. This has led to patients’ perception, perhaps unfairly, that regulators are unnecessarily delaying access to life saving therapies^2^. It is critical that regulatory frameworks constantly adapt to optimally regulate emerging technologies according to their risks, benefits, and unique properties. The article considers the optimisation of regulatory frameworks relating to the AI-based personalisation of treatment, both for classical cancer drugs and for advanced therapy medicinal products (ATMPs, medicines for human use that are based on genes, tissues, or cells as well as combinations). Most ATMPs are for cancer therapy^3,4^. ATMPs in clinical trials for both cancer and other diseases currently face substantial waiting times for approval in the US, and even longer timelines in the EU^5–7^. This situation is likely to exacerbate due to a technological paradigm shift taking place in the development and mode of adaptive use of cell and drug-based therapies. Up until now, true personalisation has been limited to the adaptation of therapeutic protocols by oncologists on a case-by-case basis for their patients, and to the creation of ATMPs from the modified cells of the individual patient. In different medical disciplines, digital approaches, often using AI, are increasingly being used for the genuine personalisation of prevention, diagnosis^8^, treatment planning and dose adaptation^9,10^, and this is relevant both for classical drug therapeutics and ATMPs.

In the area of precision diagnosis of cancer, examples include CE-marked AI-enabled products for radiological image analysis, of which an increasing number have been demonstrated to improve diagnosis compared to standard care, and some of which have demonstrated positive effect on healthcare system efficiency^11^. In the area of personalised drug treatment in cancer, digital tools for monitoring patient reported outcomes (ePRO) have been shown to increase quality of life and survival in patients with advanced lung cancer^12^, and this led in 2020 to the first reimbursed digital therapeutics solution in France^13^. This automatic monitoring tool automatically triggers alert messages to the treating oncologist, based on an automatic analysis of when patient reported symptoms fall into predefined thresholds for severity and worsening. This rule-based decision making is a simple form of AI (and is defined as AI in the proposal for an EU AI Act^14^). Through a physician-in-the-loop process, this tool enables the personalised adaptation of drug therapy in cancer. The general trend is that rule-based decision-making is over time substituted for machine/deep learning-based approaches, as more data is gathered, from on-market use and from future prospective studies^15^ These approaches can be seen in light of indexes that are already used in clinical settings for risk-adjustment of in-hospital events^16^.

In 2021 alone, more than 100 applications received by the US FDA included some aspects of AI^7,17^. The potential for the AI-guided precision design of non-personalised but disease specific ATMPs is rapidly advancing^18^. Regulatory bodies have released discussion papers addressing the regulatory approaches needed to meet these challenges^7,19^. AI approaches applied to the molecular personalisation of cell-based therapies are in development^9,20–23^. There have been previous high profile but ultimately unsuccessful BigTech excursions into precision oncology, including for drug regime planning, that have delivered some value but have substantially fallen short of their hype (e.g. the ambitious 2012 IBM Watson health project^24,25^). Is the potential for AI-enabled personalised therapeutics being over hyped? AI approaches, such as those applied in computer vision and natural language models^26^, have advanced substantially in the last decade and there is increasing consensus on and evidence for their transformative potential in medicine^7,19,27–29^. There is now the potential for much speeded development through the complementary advancements of generative models and multimodal AI models which integrate multiple data modalities, including foundation and generalist approaches, alongside decentralised approaches enabling access to large data sets^30,31^. Generalist medical intelligence (GMAI) has been proposed, which unlike previous narrow and use case specific applications of AI, could flexibly interpret combinations of medical data from electronic health records, imaging, laboratory results, genomics, and the medical literature^32,33^. A critical aspect of this new technology is the ability to interpret data of multiple types and origins^34^, a recognised need in precision oncology^35^. GMAI approaches can be repurposed to a wide range of tasks with limited manual human redesign, through drawing on knowledge of related problems^32^. This integrated information can be used not only to interpret and to generate medical image and text data but also small molecules, nucleic acid sequences and proteins^36^, including monoclonal antibodies^33^. These technologies could be used in the near term to generate patient specific precision ATMPs, if the patient specific integration of rich molecular and clinical data is combined with existing molecular genetic techniques for rapid synthesis of proteins including receptors^18,32,37,38^.

Regulatory approaches and resourcing are unlikely to keep pace with personalised therapy without a parallel paradigm shift. We compare the EU and US regulatory approaches in detail, as models of the ranges of the possible international approaches^39,40^. We describe the AI-enabled innovations that are bringing the personalisation of therapy planning and of ATMP design to the patient’s bedside and the associated regulatory challenges. The bottlenecks in current regulatory processes for these emerging technologies are described along with the range of possible solutions that could optimise safe approval pathways. These are of particular relevance to regulatory policy makers and the developers of novel precision therapies and are also of interest to oncologists and cancer researchers.

### Emerging AI-enabled personalised oncology approaches and regulatory challenges

A series of approaches for the AI-enabled personalisation of drug and cell-based therapies have been proposed (Table 1)^9,20–23^ and these face a complex regulatory environment. AI-based health care tools are regulated under medical devices law, whereas drugs and cell-based therapies are under medicines law^41,42^. There are provisions for drug-device combinations or combination products which bring medicines and devices together, however these are inadequate for the emerging complex and fluid interaction between patient data, AI and the prescription, design and adaptive dosing of medicines^9^. In this Perspective, we describe the landscape of personalised approaches in precision oncology, we explore on a case-by-case basis the regulatory readiness gap for these emerging technologies, we summarise and assess novel regulatory concepts that have been proposed to close this gap, and we suggest our own approaches and discuss their strengths and weaknesses.

**Table 1:** Digital- and AI-guided/-enabled personalised drug and cell therapy and therapy management in oncology.

#	Digital- and AI-guided/-enabled personalised drug and cell therapy and therapy management approach	Summary of transition to implementation
1	Clinical decision support (CDS) systems that facilitate therapy planning^43^	Some approaches are advanced in approval pathways and others are in early implementation. In the US, some have a non-device status not requiring specific regulatory approval^43^
2	Precision diagnostics (including companion and complementary diagnostics^59^), and AI-based multi-cancer early detection (MCED) tests^10,117^	Some approaches are advanced in approval pathways and others are in early implementation. In the US some are available under clinical laboratory waiver (US^118^)
3	Drug companion apps that personalise therapy regime management and adaptation^53,72,73^	Some approaches are in late development and clinical validation
4	personalised ATMP design^9,20–23^	Some approaches are in late development and clinical validation
5	Digital twins as an emerging concept in diagnosis and therapy - integrate near-real-time patient data management with simulation-/model-based diagnosis and therapy design and monitoring^91,93^ and generalist medical AI approaches of broad purpose medical AI^32,68^	largely theoretical/ research concepts not yet in as generalised approaches in approval pathways

### Clinical decision support (CDS) systems for healthcare providers (HCPs)

CDS provide diagnosis and/or personalised treatment suggestions, informing or driving treatment based on individual patient physiological and clinical data, e.g. combination therapy regimens^43^. In the EU, they have the regulatory classification of at least moderate risk in-vitro diagnostic devices/medical devices^44–46^. In the US, they are classified as non-medical devices if non-urgent advice is delivered with: (i) alternatives; (ii) a basis; (iii) evidence; (iv) no processing of image or signal data; (v) approaches for the prevention of automation bias^47^. Large language model (LLM)-based CDS approaches have recently been proposed including in the area of oncology^48–50^, which show impressive but variable performance^51,52^. Some LLM-based approaches are being built into clinical charting systems^53,54^. LLM-based chatbots, which claim to provide medical advice to patients, are also being made available on the EU market in a manner that is likely illegal (^29^). Any tool placed on the market with a specific claim of medical functionality, and which used an algorithm to provide specific disease related advice relevant to diagnosis or therapy is classified as a medical device and requires stringent quality and approval processes^29,55,56^.

### Specific regulatory challenges for CDS systems

Regulators, health care systems and thought leaders acknowledge both their potential (including in precision drug development and manufacturing^7^), and their inherent challenges and need for thought-out regulation^29,57,58^. GMAI and LLM system validation is challenging as they can invent data and have a near infinite range of inputs and outputs^29,57^. On these bases they are excluded from US non-device classification^29,43^.

### Precision diagnostics—companion diagnostics (CDx) and complementary diagnostics (cDx)

**Fig. 1:**
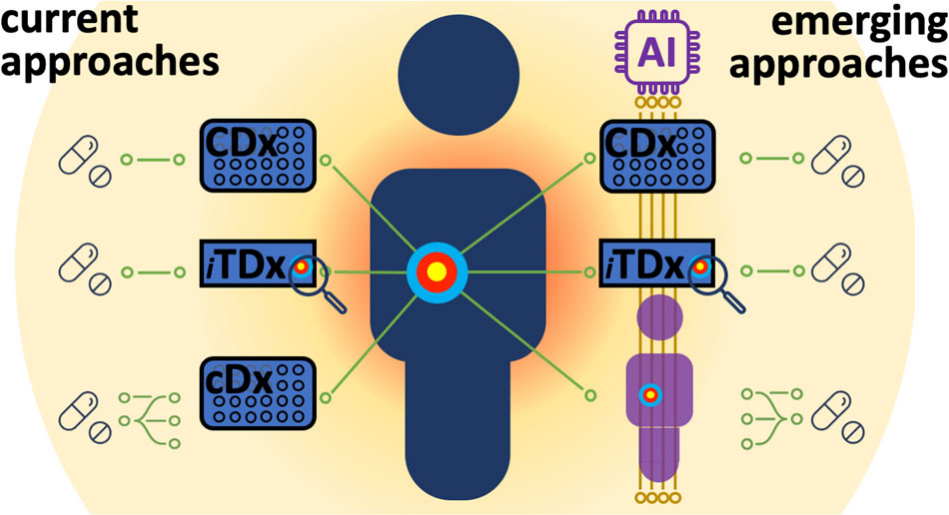
The current approaches for assay- and image-based companion/complementary diagnostics (CDx, TDx, cDx) and the emerging technologies for AI-based and information-integrative approaches. iTDx – image-based TDx.

These are in-vitro diagnostic or medical devices for deciding on medicinal product use that: (i) identify patients most likely/unlikely to benefit; (ii) identify patients likely to be at high risk of serious side effects; and/or, (iii) monitor patient response to enable treatment tuning to optimise safety and/or effectiveness^59–62^. CDx are, if applicable, obligatory for the prescription of the drug, whereas cDx provides optional additional information (e.g. on enhanced benefits in subgroups)^59^ (Fig. 1). Currently most c/CDx are chemical, genomic biomarkers and immunoassays^59,63^. In the US, the approval of the drug and c/CDx are assessed by the FDA, enabling coordinated approval^42^.

### Specific regulatory challenges for CDx and cDx

In the EU, there is a fragmented assessment through the involvement of separate drug and device regulators^64^ and regulators often cannot agree on individual approvals^65^. Trials show the potential of c/CDx in highly personalised therapy decisions^66^. Tissue-based companion diagnostics (TDx) use slide-based image analysis coupled with molecular labelling^67^ (Fig. 1). Existing regulatory frameworks can be extended to AI-based image analysis in TDx, where these are based on classical biomarkers. Increasingly, GMAI approaches are being proposed^52,68^, which are likely to use flexible combinations of non-classical biomarkers^69^. It will be challenging for non-classical biomarkers and GMAI approaches to meet explainability and transparency requirements in proposed regulations^14,47^.

### Drug companion apps

First generation cancer drug-companion apps provide simple digital support to patients undergoing cancer treatment to enhance patient understanding and adherence. They have simple diary, tracking and communications functions^53,54,70^. Some of these apps use remote monitoring for dose adaptation to side effects, but, due to the increased HCP workload they create, and due to their lack of reimbursement, they have thus far only been used in clinical trials^71–73^. These apps, depending on their precise purpose, are non-medical device apps (US and EU), medical device apps for which there is a regulatory non-enforcement approach (US)^37–39^ or low risk class medical device apps (EU)^44,46^. Newer generation apps provide specific personalised drug dosing regimens based on the patients dynamically reported or sensed side effects and some provide automated/closed-loop personalised adaptation of regimens. These have large potential in precision oncology, but implementation is more advanced in other medical disciplines, due to lower disease and drug dosing criticality e.g. FDA approved apps for diabetic home management of insulin dosage, which suggests next dose based on treatment plans^8^.

### Specific regulatory challenges for drug-companion apps

Regulatory body discussion papers acknowledge the future importance of personalised adaptive dosing^19^ but there is no clear guidance for these products. In both the US and EU, they are likely to be treated as products requiring detailed expert consultation and considered alongside the medicine’s marketing authorisation. In the EU, they can fall under the category of borderline products, i.e., complex healthcare products for which there is uncertainty over the applicable regulatory framework, and were national authorities classify them as either as medicines or as medical devices on a case-by-case basis^74^.

### Personalised ATMPs

Personalised ATMPs, manufactured for the individual patient and their disease state, are an emerging concept rather than an everyday technology. Chimeric antigen receptor (CAR) immune cell therapies are based on T-cells (CAR-T) and NK-cells (CAR-NK) generated mostly from the patient’s own immune cells, or alternatively from allogeneic effector cells. They have specific targeting through engineered receptors^75–79^. Mainstream approaches do not use AI. In the US, these are regulated through the FDA as cellular and gene therapy products^80^. In the EU, these are classified as gene therapy medicinal products, a subset of ATMPs, which require approval through the European Medicines Agency (EMA)^3,65,80^. CAR-T/NK development, manufacturing and therapeutics, and monitoring are complex^81,82^. AI-based approaches have been proposed for personalised prediction of response to CAR-T/NK therapy^83,84^ and for personalised prediction of toxicity due to cytokine release syndrome and neurotoxicity. In addition, AI enables optimising manufacture, especially through automation^77,78,85^. Optimisation of development has focused on molecular genetic engineering strategies^86–88^. Recently, AI-based bioinformatics approaches have been proposed to identify patient specific mutations, so that CAR-T/NK cells can be targeted to the patients neoantigens^20–23^. With the advent of AI-based protein and receptor design^32,33,38^, it is now more feasible that tumour mutation and antigen profiling will enable high throughput AI-based personalised CAR-T/NK cell targeting.

### Specific regulatory challenges for personalised ATMPs

Personalised ATMPs bring about a new technological paradigm, of ATMPs developed for a single purpose in a single individual, which requires a responsive regulatory framework. Although many pre-existing regulatory principles and approaches can be inherited^47^ there are also inherent new challenges^77^. FDA and EMA discussion papers acknowledge that AI may be used in de novo design of product variants in precision medicine, and in personalised treatment, but do not yet consider the design of a ATMP/receptor for an individual patient^7,19^. Personalised AI in medical diagnostics and therapeutics have up to now been entirely software based, i.e., diagnostic patient apps, CDS, AI-based digital therapeutics, and have been regulated as medical devices. This new technological paradigm is based on similar input data, i.e., the omics, physiological and clinical data of the patient. Internationally, it currently looks as though the regulatory approach applied will be a combination of inflexible frameworks, that were originally designed for fixed-product manufacture^89^, bootstrapped to new frameworks specifically designed for AI as a technology^90^.

### Digital twins as an emerging concept in diagnosis and therapy

Digital twins (DTs) are real-time representations of the patient’s physiological and clinical data that can drive an array of highly personalised simulation approaches, that could provide HCPs with highly personalised CDS for diagnosis and disease management^91,92^. These approaches could also directly drive closed loop therapy.

### Specific regulatory challenges for Digital twins

Broad concept flexible DTs have not been implemented in practice and comprehensive realisation of the DT concept is precluded under current regulatory frameworks, as these require the pre-specification of device intended purpose^39,44,93^. Flexible frameworks for health record sharing and interoperability are being developed alongside ontologies^94^, which support the DT concept^95,96^. Recent concepts for AI regulation, e.g. company-level rather than product approval, and on-market adaptivity are partially conducive to DTs^39,57,97^.

### Gaps in regulatory system readiness and why these occur

AI-based and patient data driven personalisation of diagnosis, therapy and disease management is only starting to have an impact on patient care^9^. As the step-change in the rate of development of non-medical AI, of GMAI and of approaches for the AI-based design of proteins and receptors has only been from 2022^18,28,29,32,52,68,98^, it is not surprising that regulatory frameworks are not optimally designed for these concepts and that regulatory innovation, including new approaches and associated laws and guidance are needed^29,57^. We summarise general approaches that have been proposed to address this regulatory readiness gap for AI, and propose our own in approaches, which are particularly relevant to personalisation in precision oncology, later sections of this Perspective.

The investment and effort in regulatory science, regulatory innovation and implementation need to be always tuned to the rate of advancement of new technologies, changes in healthcare delivery and changes in public and political perception of balancing of risk and benefit^39,99–102^. We propose that the reasons for regulatory frameworks being unready for advancement in technology be divided into those of: (i) out-datedness; (ii) over-extension; (iii) fragmentation; (iv) contradiction; (v) divergence; (vii) complexity, and (viii) over/under-stringency (Table 2).

**Table 2:** Our perspective on the regulatory problems particularly relevant to the efficient and safe development, approval and adoption of AI-based personalised drug and cell-based therapy in precision oncology.

Regulatory problem	Description
(i) Out-datedness	Arises when the regulatory frameworks are not updated to reflect the state of the art in technology or practice (e.g. there is no mention of AI in the EU medical devices legislation or its associated guidance^89^, perhaps as a specific AI Act has been long anticipated^39,89^).
(ii) Over-extension	Arises when regulatory frameworks are extended from earlier to later technologies without adequate adaptation to new challenges (e.g., from physical medical devices to medical software and AI^89^).
(iii) Fragmentation	Arises when there is an artificial separation of the regulatory treatment of approaches, which in practice are used together, e.g., separation of the regulation of medicines, devices, which are increasingly integrated concepts, or failure to adequately consider the interaction between technologies in medical workflows^100^ (Table 1, Fig. 1).
(iv) Contradiction	Arises when laws that apply sectors (such as medicines and devices^44,45^) have overlapping but contradictory requirements or different conformity and enforcement approaches and authorities^39,119^ than the also applicable cross-sectoral laws relating to human rights or technologies, such as AI^14^.
(v) Divergence	Arises when the regulatory framework differs substantially between or even within countries or through different interpretations of transsectorial or transregional regulatory bodies.
(vi) Complexity	Arises through badly written laws and guidance, and also if problems (i) - (v) are unresolved or badly addressed as well due to heterogeneity between member states^119^
(vii) Over/under-stringency	Arises when the requirements of the regulations or their enforcement provisions or practice are out of balance with the ratio of benefit versus risk of products, or the wishes or needs of society^39^

**Fig. 2:**
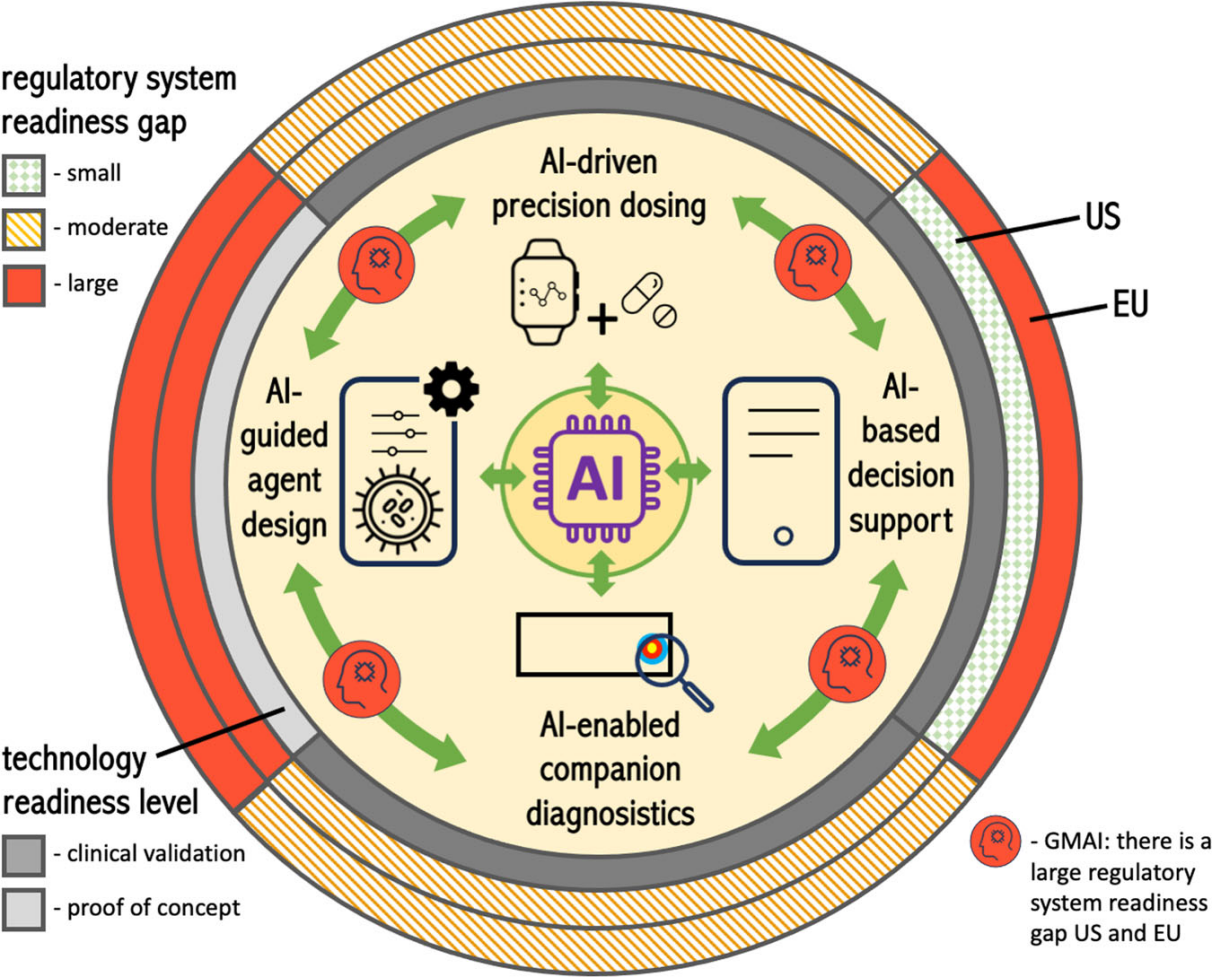
A perspective on the US and EU regulatory system readiness for AI-enabled personalised drug and cell-based therapies in precision oncology. The principal difference in regulatory system readiness is that the US FDA’s ‘non-device’ approach for AI-based CDS^43^ substantially reduces the gap between new technology and the regulatory framework readiness. The regulatory system colouring reflects current readiness only and does not reflect the potential for fast adaptability of frameworks (i.e., through greater regulatory science resources, less fragmentation, and smaller backlogs).

In our view, there are two large challenges to the appropriate regulation of AI-guided precision oncology. Firstly, the importance of the multimodal and integrative nature of developing technologies in AI/data based personalised medicine is likely to be underestimated by new laws and by regulatory bodies. Secondly, the degree of regulatory change that would be needed to address this is large^100^, is likely to be underestimated and not delivered due to institutional and political resistance, vested interests, and lack of sufficient commitment to radically pragmatic thinking. A perspective on the status quo in AI-based personalised therapies is shown in Fig. 2, in terms of their interrelatedness, their stage of development and the readiness of regulatory frameworks for them.

As AI-based interpretation of detailed real-time patient data insights enables the personalisation of diagnosis, ATMP design and adaptive dosing, then, ‘devices’ merge into continuous concepts of care, rather than discrete concepts of products^100^. Regulatory bodies are only starting, through recently released discussion papers and consultations^7,19^, to address the challenges introduced by the technologies (described in Table 1 and Fig. 2). It is likely that the problems of regulation out-datedness and over-extension can and will be addressed through careful updates to the regulations. However, taken together, the problems listed in Table 2 will have a profound impact on the development of AI-enabled personalisation of cancer therapy. The degree of contradiction between regulations may be increased through the EU and US draft proposals for the regulation of AI as a technology^14,103^, however, this may be resolved in later stages of proposal development. There may be limited political will to address divergence between international regulatory frameworks, particularly as there are major differences in the EU and US approaches to stringency^39^ (e.g., all CDS approaches are regulated as medical devices in the EU, but many are regulated as ‘non-device’ in the US^43^). The US, with its federal level regulation of the sector, is better suited to limit the fragmentation of regulatory implementation and enforcement, even given the overlapping responsibilities of different US regulators. It is claimed in the draft legislative proposal for the new EU drug regulation^104^, that it will better keep pace with technological advances and have greater clarity on the interplay between drug and cell-based therapies and devices. As of yet there is little concrete detail on how this will be achieved. Even for non-AI-based technologies, the current fragmentation of regulatory responsibility is causing major problems of approval time and supply^1^. As the EU governance structures with multiple regulatory bodies are unlikely to change, there may be no alternative than for all actors to develop approaches for maximal communication efficiency to manage the highly fragmented regulation, and to find rapid solutions to contradictions.

### Novel regulatory approaches

Long delays in the arrival of new AI-based personalised therapeutic approaches to patients are likely due to the current rate of technological development and due to the unsuitability of the existing pre-precision medicine regulatory frameworks. There is a public expectation that the medical state of the art is made available to them^39^, particularly in cancer therapy^2^. Some countries will likely develop innovative regulatory approaches that enable AI-based personalised cancer therapeutics. As the benefits of these therapies become known to the public, they will expect the regulations of their own country to allow access to these therapies. If this does not happen, public support for their national frameworks are likely to be eroded^39^. Current regulatory frameworks are a de facto blocker to AI-based personalised medicine, and transformational change of approaches is needed if this is to be resolved.

Understandably, the discussion papers of the US and EU regulatory bodies call for a relatively cautious approach^7,19^. The EMA describes all medicines related use cases of AI in precision oncology as high risk, and calls for fall-back treatment strategies in cases of technical failure^19^. For some application areas it may be necessary to adopt precautionary regulatory approaches. At the same time, it must be recognised that a precautionary approach is a brake on development and precautionary approaches themselves should be evidence-based and be balanced in terms of their risk and benefits^39^. It must be ensured that if precaution is favoured, it is precaution within maximally efficient well-resourced processes, and with the enablement of bold decision making by regulatory bodies when approval is justified. The gathering of sufficient evidence for approval of novel cancer therapeutics is time consuming but there should not be additional delay due to inefficient interagency communication.

Small incremental change will enable the technologies described in Table 1, to develop further, likely leading to a further acceleration in technological development through: (i) deep integration into clinical systems and workflows^100^; (ii) greater access to data as well as rules for larger data exchange including academic—industry partnerships; and, (iii) on-market learning. This will lead to a cycle, with the requirement of further incremental changes to regulation. Even though small, stepwise incremental change will improve regulation, transformative changes may be required to address the gaps in regulatory system readiness (Fig. 2). One such transformational change has already been made by the FDA, through classifying many CDS systems as ‘non-devices’ and hence substantially reducing the regulatory approval burden for these devices and creating a pro innovation environment for their development. We summarise transformative changes proposed in the regulatory science community, which could close the regulatory readiness gap for novel AI-based personalised therapeutic approaches in Table 3. We consider these specifically in the context of the applications in personalised drug therapies and add to these our additional proposals. We comment on the ease of introduction, the potential advantages, and possible downsides of these approaches. Some of the suggested approaches are highly innovative, and would require detailed development followed by trialling in sandboxes, according to better regulation principles^39,105^.

**Table 3:** Innovative regulatory approaches that could be applied to enable leaner regulatory clearance/approval and oversight of AI-enabled drug and cell-based therapy personalisation approaches.

The limitations of the Benefit-Risk Ratio (BRR) in personalised medicine	*Limitations of the regulatory status quo:* The BRR is a central principle in medicines and devices regulation, with approval only of products which have benefits outweighing risks^120^. For the case of some ATMPs, e.g. CAR-T/NK therapies, some experts argue that BRR is not a meaningful concept as the mechanism of action is so complex and their effectiveness relies on harnessing innate attributes of the patient’s own physiology, to a much greater degree than for traditional therapies^65^. With greater personalisation, through AI-based receptor design, monitoring and adaptive dosing, therapy will be further highly dependent on patient physiology. The whole population BRR is of limited value in guiding the oncologist or the patient, and a degree of confidence of AI prediction may be of more value than the BRR. *Innovative/agile proposals:* The implementation of N-of-1 trials to assess the BRR within a given cohort is a feasible solution^121–123^. This single-patient trial approach uses the individual person as a unit of investigation and enables individual patient BRR through measurement of efficacy and adverse events. There are many open questions^121^ on the level of background evidence before administering a new drug or cell-based therapy to a patient, how human and AI-generated ‘opinions’ are combined in this decision making^124^, and the degree to which evidence can be generalised for the overall personalisation approach, given that the drug or cell-based therapy itself or the therapy management will be highly personalised through AI. *Our perspective:* Under current regulatory frameworks^56^, it can be interpreted that evidence for a positive BRR must be demonstrated for an AI for every subset of the target population, intended purpose, and clinical indication. With the increasing potential for generalist and personalised AI, and the acknowledgement of the individual patient dependency of complex therapies, there is a need for rethink on approaches to effectively and safely integrate imperfectly-evidenced and imperfectly-grounded AI insights into decision making, even when the individual patient BRR ratio is far from certain. Here new decision-making processes, and new approaches to training clinical decision makers are needed, alongside appropriate. These could relate to allowing AI-enabled advice to have a lower position in a carefully designed clinical referral cascade, which ensures decisions in scenarios of risk are referred to highly experienced oncologists^113^. Approaches could also include allowing AI-enabled insights to be provided to tumour boards, or even allowing AI-enabled advice to have a seat on a tumour board does not surrender human thinking and final decision making^100,125^.
Breakthrough, fast track programmes and ‘airlock classifications’	*Limitations of the regulatory status quo:* Technologically novel approaches can face particular regulatory burden associated with their novelty. Novel products can, by definition, have regulatory routes based on predicate devices closed off to them^126^, may require special regulatory processes based on novelty alone^55,126^ and are likely to face bottlenecks in access to small number of regulatory approval bodies^39^. *Innovative/agile proposals:* Approaches have been proposed or trialled which convey a special status to some innovations on the basis of unmet clinical need and/or technological novelty linked to transformative potential. Some programmes provide primarily access to faster regulatory assessment^127^, others to ‘vouchers’ of financial value that can be used for faster access for other products in the manufacturers portfolio^128^. The UK’s proposed ‘airlock classification rule’ would allow temporary early market release of medium risk products followed by careful post market oversight, as if they were high-risk devices, until the risks of the device are properly understood and the permanent risk class could be defined^129^. *Our perspective:* Innovation in health care delivery is much needed, and it is critical that the regulatory system taken an open arms approach to welcoming and supporting innovators, to find flexible partnership approaches to safe market access. Regulatory and innovator co-funding of approaches like the ‘airlock classification rule’ can be for the benefit of all, but transparency and the willingness to work together are prerequisites to success, as demonstrated in the detailed findings of a US FDA pilot programme^130^.
‘Non-device’ status for lower risk CDSS	*Limitations of the regulatory status quo:* Under the traditional SaMD route of CDSS classification (including EU and IMDRF frameworks), all devices which provide any form of algorithmic decision support a medical purpose are as medical device^29^. If this advice relates in any way to diagnosis or therapy, the devices is in a risk class imposing stringent requirements on quality management, device design and changes^29^. This applies even to very simple, low risk systems which provide a range of additional points for a clinical to consider in treatment, and even when these are based on relatively banal search and information presentation algorithms. This creates a paradox, that the use of an internet search engine (which is routinely for physicians)^29^ is not regulate, but any approach to provide better suited clinical information than the search engine, is heavily regulated. *Innovative/agile proposals:* A recent change in the FDA regulation of HCP-facing CDS systems has been to define a ‘non-device’ classification, if non-urgent advice is delivered, with: (i) alternatives; (ii) a basis; (iii) evidence; (iv) no processing of image or signal data; (v) approaches for the prevention of automation bias^43^. *Our perspective:* If the FDA carries out careful market surveillance of the effects of this policy, then it may introduce enormous flexibility and innovation potential with minimal risk. This could provide benefit to patients through AI-guided medicine’s potential for fewer missed diagnosis, and benefits to health care systems through the return from better patient care enablement and more efficient care provision. The flexibility even extends to many aspects of the DT concept^93^, where these function as CDS and take the patient’s electronic health record as input. In the short-term, it is unlikely that the FDA’s approach will be taken up by the EU, where CDS are classed as at least moderate risk medical devices/in-vitro diagnostic devices. As a result EU CDS developers will face enormous disadvantages due to substantially less home market flexibility than their US counterparts, which will be further compounded by additional requirements of the EU AI Act^14^. The EU could carefully follow and learn from this approach. This risks a several year delay while evidence is collected, evaluated and policy development discussed. A better approach (which the US could also adopt) would be to immediately adopt a ‘non-device’ approach were the FDA specified qualifying criteria are met, but with the firm requirement for the provision of transparent and open real world performance monitoring data, applied to developers and implementing health care systems. This would combine the high flexibility of ‘non-device’ status with ever improving public domain data on implemented systems. It is likely that first approaches to real world performance measurement adopted by developers would be imperfect. Reaction to this, through the analysis of the inadequacy of approaches in the public research arena, and through action to enforce ‘device’ status on non-genuinely participative developers would resolve this.
On-market adaptive AI	*Limitations of the regulatory status quo:* Although a world of fixed AI and digital products is imaginable, it is hard to imagine that this is optimal. Adaptation on the basis of use, feedback from real word experience is the norm in most areas of software and AI use, where these areas interact with consumers^89,97^. Although high flexibility on changes is inappropriate to the highest risk safety critical systems (e.g., radiotherapy planning) many uses of AI in medicine are in lower risk scenarios^97^. Traditional regulatory approaches make it effectively impossible to have compliant real-time or near–real-time adaptive/continuous learning AI-enabled medical devices. *Innovative/agile proposals:* The FDA has proposed an approach for on-market adaptive AI^90^ through Predetermined Change Control Plans (PCCPs) and have introduced some of these proposals in recent guidance^39,89,97,111^. This approach allows the developer to specify a threshold/envelope of allowed changes in the AI-model, including in its performance, for which a new approval/clearance process would not be required^131^. *Our perspective:* The FDA’s guidance for PCCPs falls short of the ambition of its initial description of a framework for on-market adaptive AI-models, as changed models must be brought through standard design control processes of design justification and verification. This effectively precludes on-market adaptivity^131^. Medical device manufactures are reporting their first experience with these new approaches. As a new framework, it is likely that updates will be needed based on practical learnings^90^. Although limited in scope, nonetheless, the approach provides welcome flexibility to the US system, and EU approaches do not formally have this flexibility^89^. On-market adaptive frameworks are in the proposed EU AI Act^14,89^ and are likely to be accepted by EU regulators as the state of the art if implemented with careful oversight of real world performance^97^. Both US and EU approaches could be bolder, in allowing PCCPs for on-market adaptivity of AI-enabled devices, linked to transparent and well-designed real world performance monitoring^97^.
AI assurance, simulation approaches and independent test platforms	*Limitations of the regulatory status quo:* Current approaches for approval of all but the lowest risk class medical device are based on expert-reviewer based assessment of detailed technical dossiers for a medical device. Although this is undoubtedly necessary for the highest risk devices, it can be extremely time consuming and lead to considerable regulatory bottlenecks or the approval of lower risk AI-enabled medical devices^39^. Alternative approaches for assessment of the quality management system of the developer already exist in the EU, where for intermediate risk class medical devices, a sentinel device is assessed in detail, followed by certification of the quality management system and the ability of the developer to self-assess and release devices on the market^44^. *Innovative/agile proposals:* Precertification approaches have been piloted in the US, but as of yet, have not been introduced to general pathways^130^. Assurance of AI systems can be through the role of regulators and their assessment of the documentation of device development, quality management and clinical evidence. The FDA has introduced approaches and guidelines for using simulation/computational modelling in medical device approvals, alongside or as an alternative to traditional evidence sources, such as bench testing and clinical trials^132^. This has the potential to enhance the assessment of safety and performance, and depending on its application, to speed time to market for the monitoring of on-market adaptation of AI-enabled medical devices^133^. Related approaches are through the direct measurement of AI-based device performance through independent testing, carried out by reference laboratories and/or platforms^14,134^. Independent testing can assist in the on-market surveillance of performance, provide confidence in safety and guard against AI model drift. *Our perspective:* The approaches described above are not yet widely applied in AI-based medical devices, but as they become more established, they are likely to be also applied in drug & digital/AI-based pairings. Reference laboratories and/or platforms have large promise but also important limitations. They need to be developed with sufficient ingenuity to avoid overfitting to the artificial and unrealistic test scenarios, and proposed approaches assess AI-models in isolation from the physician-in-the-loop. AI assurance approaches must be considered as part of a group of agile approaches, and not in isolation.
Layered oversight	*Limitations of the regulatory status quo:* Current regulatory approval pathways focus on AI-enabled software being a device and focus on the approval pathway for the design of this model by the developer, at the expense of the integration of the model into a living complex interacting systems of clinical workflows in multiple different health care systems^100^. There are requirements for PMS, but these are not focused on the developer’s narrow AI-product rather than the interactive complex system that is the hospital^100,112^. *Innovative/agile proposals:* An approach that has been proposed to ensure the safe and ethical use of AI in decision support systems is to require layers of supervisory oversight. Suitably qualified oversight could be required in development, prior to implementation and through live oversight at the time of algorithm use. Additionally, supervisory oversight after implementation could be mandated. These approaches can be split into human in control, human-on-the-loop and human-in-the-loop approaches. *Our perspective:* A revolutionary and agile approach to regulation of AI in healthcare was proposed in US Algorithmic Accountability Act^103,135^, which unfortunately stalled rather than being passed into law by US Congress. Under the proposed act, approaches for the pre- and post-implementation impact assessment of algorithms by healthcare systems were introduced, and these oversight requirements would have applied to the AI-enabled drug and cell-based therapy concepts described in this Perspective. The separation of human oversight into different levels of the system and of professional responsibility^113^, on a basis of identified risks, could also enable greater flexibility for the development and on-market adaptation of these novel approaches.
Simultaneous use of overlapping independent AI models used in practice	*Limitations of the regulatory status quo:* Traditional medical device approval pathways, including for software, require fixed approaches, which are verified in a generally narrow intended purpose, and which are then, clinically validated and release on marked in a locked^89^ LLM and GMAI models, having generalist/broad scope, have properties making them less amenable to verification as fixed models, and current frameworks prevent the potential of these models in the generalist interpretation of multimodal clinical data being exploited^29^. *Our perspective on innovative/agile approaches:* A novel approach that may allow the safe and flexible application of CDS in precision oncology, and the design of personalised combination therapy regimens, would be to require at least two fully independent AI-systems to provide analysis. This would enable the HCPs in the decision-making loop to triangulate their own views and the recommendations of the two independent AI-based systems. AI-systems trained on the same data and using the same underlying AI approaches would likely share the same biases, weaknesses, and risks, but there could be a requirement for two systems that are both independent and based on different AI approaches and/or training data. This is seemingly inefficient, however, for most areas of medicine, including oncology, there are already more than one company/consortium developing solutions. Paired recommendations could provide a means for increased confidence in AI-decisions, and more rapid introduction of technologies, and could even find application in therapeutic approaches where there is no HCP in the decision-making loop, e.g., drug-companion apps, in the hands of the patient, processing real-time data to optimise dosing regimens.

### Ethical implications of AI-enabled personalised oncology

The introduction and increasing acceptance of novel digital and AI-enabled personalised medicine approaches to drug therapy, bring with it pressing ethical challenges, the foremost of which are: (i) informed patient consent for AI use in clinical decision making and patient care^106^; (ii) the threats to the physician-patient relationship; and (iii) the amplification of biases by AI systems^107,108^. The predominant treatment model in oncology, which is an authoritative ‘doctor knows best’ approach should not be allowed to be changed to an equally authoritative, but even less acceptable “machine knows best’ paradigm. Regulatory frameworks should ensure that personalisation is designed for the benefit of the patient and that it should support shared decision making within a patient-centred care approach. As such, AI implementation should always consider advancing the efficiency of care delivery alongside ethical frameworks^109^. Patients and treating physicians should be informed, through transparent labelling and other approaches, about the use of AI in diagnosis and treatment. Aspects of proposals for an EU AI Act consider these principles at a high level^14^, but future guidance for the application of AI in medicine may be required to ensure that patient centricity is achieved.

### Summary

The diagnosis and treatment of cancer patients is moving away from one-size-fits-all approaches to AI-enabled highly personalised approaches. The progression of in-pipeline concepts through to sentinel products can be anticipated to be followed by a surge of approval applications for related and next generation concepts, as an acceleration of what is already being observed^7,17^. These technologies need improved integration of AI-based concepts and regulation that is fit for purpose for these technologies^85^. Developments in foundation models in medicine^32,68^ and in digital twins of patients^91,93,110^ are likely to increase this trend, all of which will put pressure on current regulatory processes and approval pathways. As public perception and understanding of the potential of these approaches increases, it is essential that regulations are optimally developed to the domain, implemented highly efficiently and tuned based on regulatory performance^39^. We have described several approaches in Table 3, of how the regulation of AI-enabled medical devices, including personalised approaches in cancer drug therapy, can be more agile. Agility in the regulatory approach is the key, which safeguards against the regulatory framework being rapidly outstripped by as of yet unanticipated digital/AI developments. The novel approaches we summarise, and the new solutions we propose require consultation, pre-introduction impact assessment, and post hoc (ex post) analysis, and are best explored within regulatory sandboxes. US FDA approaches to enable on-market adaptive AI-based medical devices^89,90,97,111^ and the classification of many CDS use cases as non-device^43^ provide important examples of how regulation can adapt to allow greater flexibility while preserving regulator, health care system, and HCP oversight. Enhancement of hospital system quality oversight and HCP patient-level oversight may be needed in new frameworks^100,103,112–114^, but of course must be adequately resourced to avoid administrative overload that could be required for quality measurement^115,116^. The extent to which cancer patients should be allowed to make informed decisions about their choice for novel therapies, with the support of the HCP team, is already an important debate^2^, and its prominence will increase as AI-enabled personalisation progresses. Patient choice for highly personalised therapies, including those with less firmly established evidence, would likely be viewed as crossing the Rubicon by many regulators and is certainly at odds with all current paradigms of drug and device approval. As therapies become increasingly designed for the individual, their risk and benefits must be considered uniquely, and the best balance between novel therapy availability and the control of risks must be found, with consideration that the public interest is not solely in the direction of the maximum control of all risks^39^. Of course, in policy development, personalisation must never be allowed to be a false claim of manufacturers and developers to avoid the thorough evaluation of drug and cell-based therapies if these have largely the same design and delivery across large patient groups.

### Reporting summary

Further information on research design is available in the Nature Research Reporting Summary linked to this article.

### Supplementary information

REPORTING SUMMARY
